# Evaluation of the effect of calcium gluconate and bovine thrombin on the temporal release of transforming growth factor beta 1 and platelet-derived growth factor isoform BB from feline platelet concentrates

**DOI:** 10.1186/1746-6148-8-212

**Published:** 2012-11-06

**Authors:** Raul F Silva, María E Álvarez, Diana L Ríos, Catalina López, Jorge U Carmona, Cleuza MF Rezende

**Affiliations:** 1 Grupo de Investigación Terapia Regenerativa, Departamento de Salud Animal, Universidad de Caldas, Caldas, Colombia; 2 Departamento de Clinica e Cirurgia Veterinárias, Universidade Federal de Minas Gerais, Minas Gerais, Brazil

**Keywords:** Calcium gluconate, Cat, Bovine thrombin, Platelet rich plasma, Regenerative medicine

## Abstract

**Background:**

There are not reported regarding the protocols for obtaining platelet concentrates (PC) in cats for medical purposes. The objectives of this study were: 1) to describe a manual method for producing two kinds of PC in cats (PC-A and PC-B), 2) to describe the cellular population of the PC, 3) to measure and compare the effect of calcium gluconate (CG) and bovine thrombin (BT) on the temporal release of transforming growth factor beta 1 (TGF-β_1_) and platelet-derived growth factor type BB (PDGF-BB) at 3 and 12 hours post-activation and 4) to establish correlations between the cellular population of both PCs and the concentration of growth factors (GF). Blood samples were taken from 16 cats for complete blood count, plasma collection and PC preparation. The PC were arbitrarily divided into two fractions, specifically, PC-A (lower fraction) and PC-B (upper fraction).

**Results:**

The platelet counts were significantly different (P<0.05) between the PC and whole blood but not between the PC fractions. The TGF-β_1_ concentration efficiencies for PC-A and PC-B activated with CG were 42.86% and 46.54%, and activated with BT were 42.88% and 54.64%, respectively. The PDGF-BB concentration efficiencies for PC-A and PC-B activated with CG were 61.36% and 60.61%, and activated with BT were 65.64% and 72.12%, respectively. The temporal release of GFs showed no statistically significant difference (P>0.05) between the activating substances at the time or for any PC fraction.

**Conclusions:**

Whatever the activation means, these preparations of cat PC provide significant concentrations of platelets and GFs for possible clinical or experimental use.

## Background

The clinical use of autologous platelet concentrates (PC) for regenerative aims in veterinary medicine has focused on the field of equine medicine and surgery [1]. To date, there are studies indicating the clinical utility of several types of PC in horses with musculoskeletal disease [2-4] and limb skin wounds [5]. PC has also been evaluated as a coadjutant substance (alone or in combination with biomaterials) in canine models of bone regeneration [6,7] and osseous integration [8].

The rationale for the use of PC stems from the fact that platelets (after being activated) release substantial amounts of growth factors (GF) and other molecules that modulate inflammation and tissue repair [9]. Platelets store at least 7 GFs directly implicated in wound healing and tissue homeostasis. However, transforming growth factor beta 1 (TGF-β_1_) and platelet-derived growth factor type BB (PDGF-BB) are mainly contained in platelet alpha granules. These proteins are essential, among other biological actions, for extracellular matrix (ECM) deposition, angiogenesis and cell migration [10].

Recently, a equine PC classification has been proposed for improving the knowledge on the kind of cells and growth factors (GF) that are being used in horses with natural disease. Briefly, liquid PC (those in what a anticoagulant is used for their elaboration) could be classified in pure-platelet rich plasma (P-PRP) and leukocyte-platelet rich plasma (L-PRP). P-PRP is characterized by a platelet count slightly higher (1.3-4x) to the basal count of platelets in whole blood and the leukocyte count is lower than or similar to the leukocyte count in whole blood. On the other hand, L-PRP has increased platelet (5 fold) and leukocyte (3 fold or more) counts when compared to whole blood [1].

Either, P-PRP or L-PRP have been used clinically alone or after activation with several substances, such as calcium salts and thrombin (either from a bovine or autogenous source), among others. Calcium is an important second messenger in the platelet activation cascade because calcium mediates the characteristic platelet activation responses, such as shape change, granule secretion and aggregation [11]. The activation of platelets by most stimulatory agents leads to an increase in the concentration of cytosolic calcium (Ca2+). Platelet responses that are directly dependent on an increase in (Ca2+) include integrin activation, release of the second wave mediators, ADP and thromboxane A2 (TxA2), and the expression of platelet procoagulant activity mainly by the generation of thrombin [12].

Thrombin is the most potent platelet activator. Thrombin produces fibrin generation from fibrinogen and also contributes to the formation and consolidation of the hemostatic plug [13]. This protein generates signaling cascades within the platelets by interacting with two membrane receptors coupled to G proteins belonging to the family of protease-activated receptors (PAR) and known as PAR1 and PAR4 [14].

Cats can develop various chronic musculoskeletal problems and suffer serious traumatic injuries [15] that may be susceptible to treatment with platelet concentrates, as happens with humans [16-19] and horses [2,20,21]. PC could also be used as a coadjutant biomaterial in orthopedic surgery in cats [22]. However, after reviewing the literature, we have not found any reports regarding the protocols for obtaining PC in cats for regenerative medicine purposes. We also did not find any published data about the concentration and temporal release of GF (such as TGF-β_1_ and PDGF-BB) from feline PC activated with calcium salts or thrombin.

The aims of this study were 1) to describe a manual method for producing two kinds of PC in cats, PC-A and PC-B, 2) to describe the cellular population of the PC obtained, 3) to measure and compare the effects of calcium gluconate (CG) and bovine thrombin (BT) on the temporal release of TGF-β_1_ and PDGF-BB from feline PC at 3 and 12 h post-activation and 4) to establish possible correlations between the cellular population present in the PC and the concentration of growth factors.

## Results

### Hemogram

The packed cell volume, counts for PLT, absolute counts for MON and GRA and MPV and PDW were significantly different (P<0.05) among the two PC and the whole blood. However, the platelet parameters did not differ significantly between each PC. The WBC counts were significantly different (P<0.05) among the whole blood, PC-A and PC-B. The situation was the same for the relative counts for LYM, MON, GRA and EOS. However, the absolute count for LYM was similar between the whole blood and PC-A but differed statistically (P<0.05) for PC-B (Table 1).

**Table 1:** Cell counts / μL in whole blood, portions A – B of autologous platelet concentrates (PC)

**Variable**	**Whole Blood**	**PC-A**	**PC-B**
Platelets x 10^3^/μL	349.35 (28.64)	639.62 (44.30) a	605.34 (41.72) a
PCV %	33.73 (0.99)	2.23 (0.30) a	1.32 (0.17) a
RBC x 10^3^/μL	7151 (282)	425 (26,8) a	323 (35,9) b
WBC x 10^3^/μL	7.91 (0.75)	5.19 (0.65) a	2.24 (0.35) b
Lymphocytes x 10^3^/μL	3.22 (0.26)	3.44 (0.34) a	1.84 (0.31) b
Lymphocytes %	42.80 (3.50)	71.19 (4.31) a	79.25 (2.93) a
Monocytes x 10^3^/μL	1.09 (0.02)	0.35 (0.01) a	0.00 (0.00) a
Monocytes %	1.28 (0.23)	0.39 (0.17) a	0.00 (0.00) b
Granulocytes x 10^3^/μL	4.18 (0.53)	1.52 (0.30) a	0.40 (0.07) a
Granulocytes %	51.54 (3.28)	26.14 (3.83) a	20.13 (2.97) b
Eosinophils x 10^3^/μL	0.40 (0.08)	0.20 (0.07) a	0.00 (0.00) a
Eosinophils %	4.46 (0.57)	2.35 (0.89) a	0.00 (0.00) b
MPV (fL)	8.96 (0.38)	11.58 (0.46) a	11.57 (0.44) a
PDW %	15.51 (0.38)	16.66 (0.34) a	16.88 (0.28) a

Data presented as means (standard error). PCV, hematocrit; **RBC, red blood cell**; WBC, leukocytes; MPV, Mean Platelet Volume; PDW, Platelet Distribution Width. ^**a-b**^Different letters denote statistically significant differences between rows (PC-A vs. PC-B) by SNK test (P<0.05).

### Total protein concentration

The total protein concentration was significantly lower (P<0.05) in both PC in comparison with plasma. However, this parameter did not differ between each PC (Table 2).

**Table 2:** Concentration of growth factors in plasma, supernatants of PC portions at 3 and 12 hours

**Activating substance**	**Variable**	**Blood component**				
		**Plasma**	**PC-A (3 h)**	**PC-A (12 h)**	**PC-B (3 h)**	**PC-B (12 h)**
Calcium gluconate	TGF-β_1_ (ng/mL)	7.99 (1.59) a	23.97 (2.03) b	24.79 (2.22) b	26.03 (2.23) b	25.70 (2.43) b
	PDGF-BB (pg/mL)	365.89 (91.02) a	1571.56 (204.5) b	1420.32 (139.4) b	1552.23 (188.1) b	1400.75 (131.5) b
	Total protein (mg/mL)	52.93 (1.15) a	44.88 (0.93) b	46.70 (1.36) b	46.21 (1.54) b	46.67 (1.71) b
	TGF-β_1_ (ng/mg of TP)	0.15 (0.03) a	0.53 (0.04) b	0.53 (0.05) b	0.57 (0.05) b	0.56 (0.52) b
	PDGF-BB (pg/mg of TP)	6.91 (1.82) a	35.02 (4.12) b	30.41 (2.50) b	33.59 (3.66) b	30.01 (3.08) b
Bovine thrombin	TGF-β_1_ (ng/mL)	7.99 (1.59) a	23.98 (2.43) b	28.22 (2.59) b	30.56 (4.47) b	35.74 (4.18) b
	PDGF-BB (pg/mL)	365.89 (91.02) a	1681.15 (188.3) b	1687.40 (138.8) b	1847.15 (208.3) b	1683.16 (122.7) b
	Total protein (mg/mL)	52.93 (1.15) a	42.29 (1.37) b	45.05 (2.25) b	43.20 (2.35) b	44.92 (2.15) b
	TGF-β_1_ (ng/mg of TP)	0.15 (0.03) a	0.57 (0.06) b	0.65 (0.07) b	0.69 (0.08) b	0.79 (0.09) b
	PDGF-BB (pg/mg of TP)	6.91 (1.82) a	39.75 (4.38) b	37.45 (3.49) b	42.77 (5.29) b	37.47 (3.11) b

TGF-β_1=_ transforming growth factor beta 1, PDGF-BB= platelet-derived growth factor type BB. Data presented as means (standard error). Different letters denote statistically significant differences between rows (Plasma vs. PCs) by SNK test (P<0.05).

### Transforming growth factor beta 1 concentration

The concentrations for TGF-β_1_ were similar between each PC but significantly higher (P<0.05) in comparison with the plasma. Both activating substances presented a similar effect on the release of this growth factor over time (3 and 12 h) (Table 2). When the TGF-β_1_ concentrations were compared at 3 and 12 hours between each activating substance for each PC fraction, no statistically significant differences were found. No significant differences were observed when were compared the concentrations of TGF-β1 (pg/mg of TP) in each PC (activated with either BT or CG).

### Platelet-derived growth factor BB concentration

The concentrations for the PDGF-BB were similar between each PC but were significantly higher (P<0.05) in comparison with the plasma. Both activating substances presented a similar effect on the release of this growth factor over time (3 and 12 h). However, a significant difference was observed (P<0.05) in the concentrations of PDGF-BB (pg/mg of the TP) when the PC-B fraction was activated with BT at the 12 hours (Table 2).

### Correlations

No statistically significant correlations were found between the evaluated parameters.

### Collection efficiency

The platelet collection efficiencies were 26.16% and 24.75% for PC-A and PC-B, respectively, thereby giving a combined efficiency for the two portions of 50.91%. The platelet concentrations were 183.10% and 173.28% higher with respect to whole blood for PC-A and PC-B, respectively. The growth factor collection efficiency at 3 and 12 h for each activating substance is presented in Table 3.

**Table 3:** Concentration efficiency of growth factors in the PC supernatants at 3 and 12 hours

**Activating substance**	**Variable**	**Blood component**					
		**PC-A (3 h)**	**PC-A (12 h)**	**PC-B (3 h)**	**PC-B (12 h)**	**PC-A+B (3 h)**	**PC-A+B (12 H)**
Calcium gluconate	TGF-β_1_ (%)	42.86	44.32	46.54	45.95	89.40	90.27
	PDGF-BB (%)	61.36	55.46	60.61	54.69	121.97	110.15
Bovine thrombin	TGF-β_1_ (%)	42.88	50.46	54.64	63.90	97.52	114.36
	PDGF-BB (%)	65.64	65.88	72.12	65.82	137.76	131.70

TGF-β_1=_ transforming growth factor beta 1, PDGF-BB= platelet-derived growth factor type BB.

## Discussion

This research describes a simple centrifugation manual (tube method) protocol to obtain PC from feline blood, thereby concentrating the growth factors such as TGF-β1 and PDGF-BB for experimental or clinical application in this species. The protocol described here presents the advantage that the PC is easily obtained with one centrifugation step with a small volume of blood. This last situation is important in feline practice because the volume of blood required to obtain PC for clinical application could be a limiting factor, especially in pediatric patients. To note, both PC obtained in this study could be classified as P-PRP.

We have not found any published studies about preparation of PC (either P-PRP or L-PRP) for clinical use in cats for regenerative medicine purposes. However, we did find information regarding manual methods for concentrating feline platelets for evaluating *in vitro* the effect of aggregating and anti-platelet substances in this species. In those studies, sodium citrate 3.8% was used as an anticoagulant. The studies included different centrifugation protocols ranging between 150–200 g and times of centrifugation ranging from 10–20 min [23-29]. These studies did not present data on the number of platelets concentrated or other hematological information with respect to the characteristics of those PC. For this reason, this study presents novel information about feline hematology with potential applications of PC for regenerative medicine purposes in cats.

The cellular characteristics obtained in both PC differed only in the highest concentration of LYM found in PC-A. This finding could suggest that each PC could display different biological effects mediated by this kind of cell when used clinically. This assumption can suggest a difference in clinical application characteristics of each portion due to the important regulatory effect of leukocytes in the healing process [30]. Particularly, lymphocytes are one major source of granulocyte-colony stimulating factor, granulocyte macrophage-colony stimulating factor, interleukin-1 and tumor necrosis factor alpha. These proteins have functions related to wound healing because they increase the activity of neutrophils and monocytes and promote the proliferation of keratinocytes and fibroblasts. All these actions are important in the inflammatory phase of wound healing [31]. However, this suggested mechanism is only an assumption, and additional experimental work is necessary to evaluate this hypothesis.

The platelet collection efficiency was low (26.16% for PC-A and 24.75% for PC-B) in this study. This low efficiency is one of the main characteristics of manual methods (tube) to concentrate platelets in humans [32] and horses [33]. However, no other published results have been found for cats to compare with these results. The platelet collection efficiency obtained in this study could be sufficient (in terms of the concentration of platelets) to produce biological effects because the high concentrations of platelets could suppress cell viability and proliferation [34]. This concept is still controversial and should be the subject of future studies in cats. One limitation, with the platelet count of this study was that blood smears were not made to ensure no platelet clumping, this could be a potential limitation because clumping would influence the platelet counts.

The MPV represents the average size of the platelets, and PDW is an indicator of variation in the size of the platelets. The MPV and PDW values for automated hematology instruments would be increased during platelet activation [35]. The MPV and PDW values were lower in whole blood that in either PC. However, these platelet activation related parameters remained in a normal rank in both PC [36,37]. These values would indicate that the methodology used to obtain the PC in cats did not produce platelet activation. This concept is important if we consider that an effective method for concentrating platelets should first focus on obtaining functional and non-activated platelets instead of concentrating a large number of platelets [38]. In the light of procoagulant properties of feline platelets, this statement must be interpreted with caution and could be a limitation of this study.

Autologous platelet concentrate preparation involves a series of centrifugation and separation cycles for concentrating the platelets without inducing premature activation. The size and weight of the blood cells and the relative forces (g) and time of centrifugation are the factors that determine the cellular and molecular characteristics of a PC. Studies in dogs [39], rabbits [40], pigs [41], horses [33] and humans [42] describe protocols with two rounds of centrifugation. One of these episodes is always greater than 240 g, and one of the centrifugation times is greater than 10 minutes (except for horses). In the case of cats, the PLT can be concentrated by a soft spin (85 g) and short time (6 minutes). This methodological difference between species to obtain PC may be due to the morphological characteristics of cat platelets, such as higher diameter (2–6 μm) [43] and mean platelet volume (8.6-14.1 fL) [36].

As described in a study in horses [33], samples were kept in incubation at room temperature (20–22°C) for two hours after activation. In our case (incubation at 37°C), within two hours after activation, we did not yet have complete formation of a fibrin clot in most of the PC samples. For this reason, the cat samples were left in incubation for another hour (three hour, total incubation time). This difference in time required for the formation of fibrin clots can be due additionally to the peculiar morphology of the platelets of both species and to the cellular count of the PC and whole blood of each species. The platelet counts in whole blood and PC in horses [33], are lower than those found in the cats in the present study; in particular, the PC show three-fold higher platelet concentrations in cats than in horses. This high concentration of platelets in cat PC compared with the results reported in horses [33] can lead to longer incubation times and/or higher doses of activating substance to obtain clots in cat PC.

It is reported a TGF-β_1_ mean concentration of 21.48 ± 8.948 ng/mL in serum samples taken from 12 cats [44]. This concentration was similar to the concentrations found in the supernatants of both PC but higher than the plasma TGF-β_1_ concentration in this study. This discrepancy was caused by premature platelet activation. Serum differs from plasma in that the bulk of the fibrinogen has been removed by conversion into a fibrin clot together with the platelets that have either been physically bound in the fibrin matrix or activated to form aggregates or both [45]. This finding implies previous blood clotting and therefore platelet activation and release of the growth factors contained in the platelet alpha granules, including TGF-β_1_.

We were not able to find any reports on feline PDGF-BB plasma concentration. To the best of our knowledge, this study is the first time that an ELISA human kit for PDGF-BB was used to measure this protein in blood components from cats. However, it is reported that both human and cat PDGF-BB presented high peptide sequence homology [46]. A similar finding has also been noticed between human and equine PDGF-BB [47].

Once the PC is prepared, platelet activation (exogenous or endogenous) may be important to maximize growth factor release. Various substances have been described for the exogenous activation of PC, including thrombin [48], batroxobin [49], collagen type I [50] and calcium chloride [2], among others. The substances most frequently used to activate PC for clinical purposes are thrombin and calcium salts. The use of topical bovine thrombin has been reported in humans to cause the formation of antibodies against the coagulation factor V, prothrombin and thrombin [51]. Reports in mice show the formation of antibodies against autologous clotting factors and the induction of autoimmunity with features characteristic of systemic lupus erythematosus, including antibodies against nuclear antigens, native DNA, double-stranded DNA and cardiolipin [52]. For this reason, the clinical use of bovine thrombin as a platelet activator in feline medicine should be carefully studied. Reports in humans [53] and horses [54] investigated the use of autologous thrombin obtained by the addition of calcium gluconate to the plasma. PC activation with autologous thrombin might provide another option for clinical practice in cats, and the probability of immunological reactions would be reasonably smaller. In addition, the results of this study reported that CG has an action comparatively equipotent to BT. These reasons suggest the use of CG to induce gelation of the PC for clinical purposes in cats.

## Conclusion

In conclusion, the methodology presented in this report permits the concentration of platelets potentially suitable for clinical and experimental use in feline medicine. The presence of significantly higher amounts of growth factors in the supernatant of PC compared to plasma indicates that PC can be used as a source of growth factors. The presence of high numbers of lymphocytes in PC-A may indicate different clinical applications for each PC. The temporary release of the growth factors indicates that the bulk of the growth factors are released during the first 3 hours after PC activation. The lack of differences in growth factor concentrations indicates that for this concern, PC activation can be made with either calcium gluconate or thrombin. The clinical value of the data reported here requires further evaluation in clinical settings.

## Methods

The ethics committee of animal research of Federal University of Minas Gerais approved this study. Owners of the cats included were informed of the nature of the research and signed an authorized consent prior sedation and blood collection.

### Animals

Sixteen mixed breed cats from local owners were used, specifically, eight males and eight females with an age range between 18 to 108 months and mean body weight of 3.4 kg that were clinically healthy at the time of blood collection. Cats with a basal platelet count less than 300 ×10^3^ PLT/μL were not included (normal basal minimum count) [55].

### Preparation of platelet concentrates

After the cats were sedated (xylazine, 0.5 mg / kg + butorphanol 0.1 mg / kg, IM), blood was collected by puncturing the jugular vein with a 21 G butterfly catheter. The blood samples were collected into two 8.5 mL tubes containing 1.5 mL of ACD-A solution (trisodium citrate 22 g/L, citric acid 8 g/L and dextrose 24.5 g/L) (Becton Dickinson and Company Vacutainer, New Jersey, USA). Seven mL of whole blood was collected per tube.

To obtain both PC, the blood was centrifuged (Hettich Rotofix 32A, Tuttlingen, Germany) at 85 g for 6 minutes. The plasma derived from the blood centrifugation was arbitrarily divided into two equal fractions, namely, PC-A and PC-B. Platelet concentrate-A (lower fraction) was considered as the first 50% plasma fraction near to the packed cell volume (PCV), and PC-B (upper fraction) represented the 50% remaining plasma.

### Hemogram

Samples from whole blood and both PCs were analyzed using an automated counting device by volumetric impedance (MEK-6400, Nihon Kohden, Tokyo, Japan). Each sample was analyzed in duplicate. The hematological parameters determined were PCV, platelet count (PLT/μL), red blood cell count (RBC) and white blood cell count (WBC/μL). The absolute (cell/μL) and relative (%) counts for lymphocytes (LYM), monocytes (MON), granulocytes (GRA) and eosinophils (EOS) were determined. The platelet activation associated parameters, mean platelet volume (MPV fL) and platelet distribution width (PDW %) were also analyzed.

### Activation of platelet concentrates

One milliliter of PC-A and 1 mL of PC-B were divided into 500-μL aliquots and activated with the addition of either 50 μL of calcium gluconate 10% (Ropsohn Therapeutics Ltda, Bogotá, Colombia) or 50 μL of a bovine thrombin solution (BioPharm Laboratories LLC, Bluffdale, Utah, USA) containing 500 IU/mL. After activation, the samples were kept at 37°C in an incubator. One hundred fifty μL of supernatant (after spontaneous clot retraction) of each PC were collected at 3 and 12 hours after PC activation. In addition, plasma samples were obtained by centrifugation of the whole blood at 1500 g for 15 minutes. The supernatants of the activated PC (platelet gel) and plasma samples were aliquoted and frozen at −82°C for subsequent determination of the TGF-β_1_ and PDGF-BB concentrations.

### Determination of total protein

The total protein concentration (TP) was measured in both PC and plasma with the biuret method (Biosystems, Barcelona, Spain) in a semiautomatic chemistry analyzer (RT-1904CV, Shenzhen, China).

Determination of the concentration of transforming growth factor beta 1 and platelet-derived growth factor type BB

The concentrations of both GF in both PC and plasma were determined by an ELISA of development with antibodies to human TGF-β_1_ (Human TGF-beta 1 DuoSet, DY240E, R&D System, Minneapolis, MN, USA) and human PDGF-BB (Human PDGF-BB Duoset DY220E, R&D Systems, Minneapolis, MN, USA). ELISA was performed according to the instructions of the manufacturer. The mean detection sensitivity was 4.6 pg/mL for TGF-β_1_ and less than 15 pg/mL for PDGF-BB. Measurements of the concentrations of both GF were performed in duplicate at 450 nm.

### Statistical analysis

All the evaluated parameters presented normal distribution (Shapiro-Wilk test, P>0.05) and were presented as means and mean standard error. Comparisons between the groups were performed using a one-way ANOVA, and post-hoc par-wise comparisons were performed using a Student-Newman-Keuls (SNK) test. A paired *t*-test was used to compare the temporal release of both GF at 3 and 12 h. Correlations between the GF concentrations and the cellular data were determined using a Pearson test. A value of P<0.05 was accepted as statistically significant for all of the tests.

### Collection efficiency

The platelet collection efficiency was determined using the following formula: (PC volume × platelet count in the PC / whole blood volume × platelet count in whole blood) × 100 [56]. The GF concentration efficiency was determined using the formula (GF concentration × PC volume / plasma GF concentration x whole blood volume) × 100 [32].

## Abbreviations

CG: Calcium gluconate; BT: Bovine thrombin; EOS: Eosinophils; GF: Growth factors; GRA: Granulocytes; LYM: Lymphocytes; L-PRP: Leukocyte-platelet rich plasma; MON: Monocytes; MPV: Mean platelet volume; PC: Platelet concentrates; PCV: Packet cell volume; PDGF-BB: Platelet-derived growth factor type BB; PDW: Platelet distribution width; PLT: Platelets; P-PRP: Pure-platelet rich plasma; TGF-β_1_: Transforming growth factor beta 1; TP: Total protein.

## Competing interests

Authors declare no competing interests related with this manuscript.

## Authors’ contributions

RFS conceived of the study, performed the laboratory tests, performed the statistical analysis and participated in the drafting of the manuscript. MEA, DLR and CL, collected samples and performed the laboratory tests. CMFR participated in the design and participated in the drafting of the manuscript. JUC coordinated the study, participated in the design and harmonized the drafting of the manuscript. All authors read and approved the final manuscript.
